# Relevance and utility of the *in-vivo* and *ex-vivo* optical properties of the skin reported in the literature: a review [Invited]

**DOI:** 10.1364/BOE.493588

**Published:** 2023-06-21

**Authors:** Kerry Setchfield, Alistair Gorman, A. Hamish R. W. Simpson, Michael G. Somekh, Amanda J. Wright

**Affiliations:** 1 Optics and Photonics Research Group, Faculty of Engineering, University of Nottingham, NG7 2RD, UK; 2 School of Engineering, University of Edinburgh, EH8 9YL, UK; 3 Department of Orthopaedics, Division of Clinical and Surgical Sciences, University of Edinburgh, EH8 9YL, UK

## Abstract

Imaging non-invasively into the human body is currently limited by cost (MRI and CT scan), image resolution (ultrasound), exposure to ionising radiation (CT scan and X-ray), and the requirement for exogenous contrast agents (CT scan and PET scan). Optical imaging has the potential to overcome all these issues but is currently limited by imaging depth due to the scattering and absorption properties of human tissue. Skin is the first barrier encountered by light when imaging non-invasively, and therefore a clear understanding of the way that light interacts with skin is required for progress on optical medical imaging to be made. Here we present a thorough review of the optical properties of human skin measured *in-vivo* and compare these to the previously collated *ex-vivo* measurements. Both *in-vivo* and *ex-vivo* published data show high inter- and intra-publication variability making definitive answers regarding optical properties at given wavelengths challenging. Overall, variability is highest for *ex-vivo* absorption measurements with differences of up to 77-fold compared with 9.6-fold for the *in-vivo* absorption case. The impact of this variation on optical penetration depth and transport mean free path is presented and potential causes of these inconsistencies are discussed. We propose a set of experimental controls and reporting requirements for future measurements. We conclude that a robust *in-vivo* dataset, measured across a broad spectrum of wavelengths, is required for the development of future technologies that significantly increase the depth of optical imaging.

**Figure:**
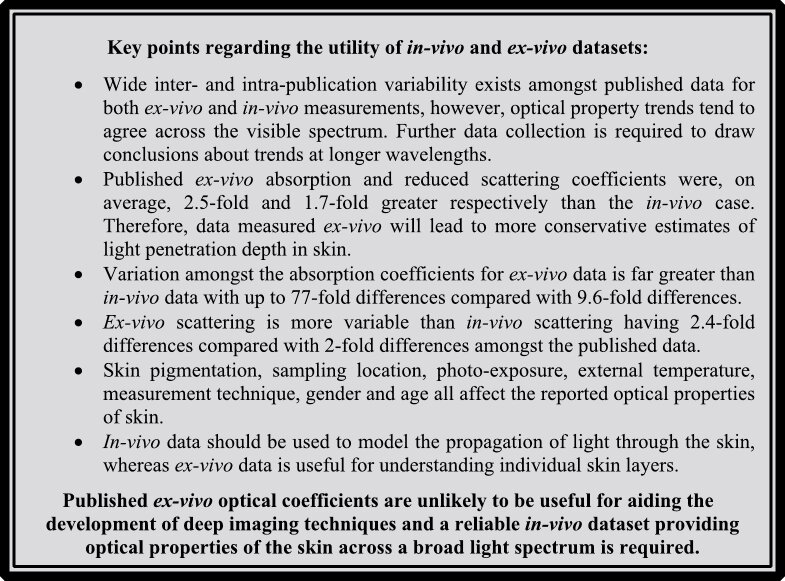


**Figure:**
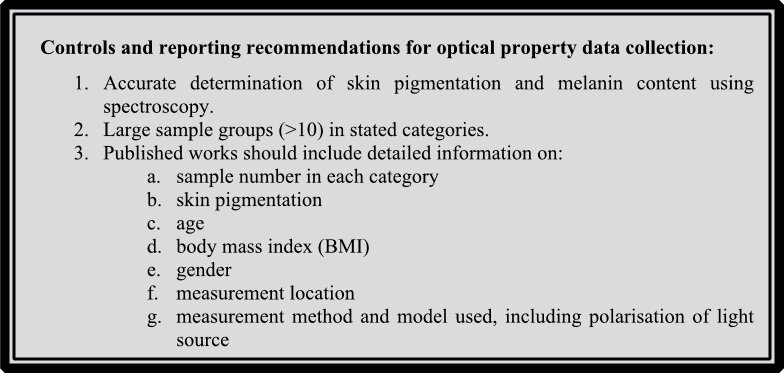


## 1. Introduction

Low-cost non-invasive deep imaging *in-vivo* with high resolution are long held aspirations of medical imaging. If this could be achieved with a non-ionizing modality such as light, it would be highly beneficial. There has been a recent surge in publications describing the optical properties of tissues both *in-vivo* and *ex-vivo* from multiple animal sources but not focussed on the skin, with more than 200 papers being published annually since 2011 [1,2]. Martins *et al.,* emphasise the importance of reliable experimental *in-vivo* data.

If we want to image inside the human body using light, skin is the first barrier to light transmission. Skin is a multi-layered heterogeneous mix of scatterers and absorbers which affect our ability to image beyond it. Photons encounter approximately 10 scattering events per millimetre when travelling through the skin, making focussing, and collecting light increasingly more difficult the deeper below the surface we image. Technologies currently available for imaging the layers of the skin, are limited in depth and therefore for skin diseases, like skin cancer, biopsies are still required for reliable diagnosis [3]. Optical coherence tomography (OCT), reflectance confocal microscopy and multiphoton microscopy have become advanced enough for clinical imaging, diagnosis and monitoring of skin, however, OCT is the only method currently used to image deeply (up to 1 mm in the skin) [4]. To advance imaging depth and methodologies the optical properties of the skin must be accurately determined. Light transmission through the skin is further complicated by skin thickness varying with location on the body. For example, the epidermis is thickest on the soles of the feet (∼660 µm), and thinnest in the eyelids (∼130 µm). The dermis, however, is thickest on the back (up to 4 mm) and thinnest on the eyelids (∼ 215 µm). Light scattering and absorption also differ at various locations due to the heterogeneous nature of skin composition [5–7].

**Fig. 1.**
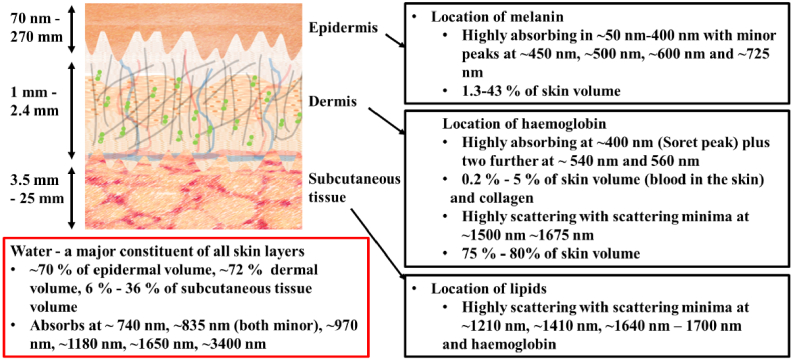
Schematic of the three different layers of the skin detailing the chromophores and major constituents of each layer.

The skin is generally considered to be stacked layers of epidermis, dermis, and subcutaneous tissue each with varying thickness and different optical characteristics associated within each layer [8] (Fig. 1). There is a wide variation in the literature for the optical properties of these different layers and the skin as a whole. It is, therefore, difficult to decide which data in the literature are the correct ones to use, especially if these values are being used to inform the design of new optical instrumentation.

The purpose of this review is two-fold: 1) to extract the reported skin optical properties systematically from the literature comparing data collected *ex-vivo* with *in-vivo* and 2) to determine the significance of these values in the context of *in-vivo* measurements. As such, the aim of this paper is to put the variation in the published figures into context, concentrating on the published *in-vivo* data for the absorption and scattering coefficients, looking at the effect of the variation in these on the distance photons will travel before being completely scattered, and determining the percentage of light reaching the different layers of skin over a range of wavelengths. It should be noted that polarisation of light will change as it propagates through tissue, however, the overwhelming majority of papers used for this review do not mention the effect skin has on polarisation and we have therefore been unable to comment on this effect. We will consider the depth at which there might be enough light to image. Our findings indicate limitations in the published optical properties, and we discuss the relevance of these values for deep imaging.

Where published data has been analysed for this review, these data have been pooled and average coefficients used regardless of method to determine the average optical properties for *in-vivo* and *ex-vivo* data. Other publications attempted to control for variations such as method used, or equations used by authors to determine the optical properties of the skin. However, despite picking data based on similarities and confidence in the data published, vast variability was still evident. Therefore, we have chosen to take an average of all data acquired from the literature, separated only by being *ex-vivo* or *in-vivo* and location, where stated, to determine trends with wavelength.

## 2. Absorption, scattering and anisotropy of photons in the skin

To understand the interaction of light with skin and to determine how light propagates through the different layers of skin it is important to consider both the scattering and absorption of photons. Photons can be destroyed by inelastic scattering and electronic transitions resulting in absorption. Typically, when absorption occurs the energy of the photon is lost in the form of heat. To characterise absorption, we use the absorption coefficient *µ_a_* which has units of length^-1^ and describes how the intensity of a beam is reduced due to absorption as it propagates through a material where,

(1) $$I_{out}=I_{in}e^{-\mu_{a}l}$$

*I_in_* is the intensity entering the absorber, *I_out_* is the intensity exiting the absorber and *l* is the thickness of the material.

Scattering occurs when the direction of a photon is changed by the presence of a scattering centre within a sample. In this article we are concerned only with elastic scattering where the energy of the photon is conserved during the scattering process and hence the wavelength of the light is unchanged. In a medium containing a number of scattering centres we can define a scattering coefficient, $\mu_{s}=n\sigma_{s}$ , where *n* is the number of scattering centres per unit volume and *σ_s_* is the scattering cross sectional area. Like the absorption coefficient, the scattering coefficient, *µ_s_*, has units of length^-1^ and provides a measure of how many scattering events take place over a unit distance or how rapidly photons change direction as a beam propagates through a sample.

To fully understand the impact of a scattering event it is also important to consider the angle a photon is scattered by and hence the anisotropy factor, *g*, which is the average cosine of the scattering angle ranging from -1 to +1. Anisotropy and scattering coefficients are generally combined to give the reduced scattering coefficient *µ’_s = _µ_s_(1 - g)*, providing more information than the scattering coefficient alone regarding how light propagates through a tissue [9]. The reduced scattering coefficient combines the effect of the number of scattering events and the severity of the scattering, so they can be combined in a single parameter. This approximation is valid for a large number of scattering events, for weak scattering the parameters are not separable. For an isotropic scatterer *g* tends to 0 and *µ’_s_* tends to *µ_s_*, and for a highly forward scattering material *g* tends to 1 and *µ’_s_* tends to 0 meaning that although scattering is occurring it is having very little impact on the overall losses of the beam. For *g* < 0 the photons are predominantly backscattered and *µ’_s _*> *µ_s_*, effectively increasing the rate at which photons are lost.

**Fig. 2.**
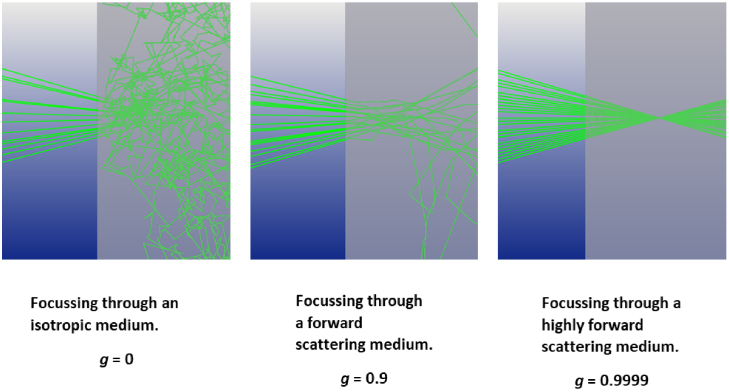
Effect of ‘g’ on focus; µ_s _= 0.2 mm^-1^, n = 1, µ_a _= 0, f-number of lens = 1.7 Images generated via Monte Carlo simulation with 25 photon paths shown for illustration purposes.

Figure 2 shows the effect that *g* will have on focussing. It is tissue dependent and ranges fm 0.69 in the human uterus at 635 nm to 0.97 at 633 nm for human blood [10]. For skin, *g* tends to be between 0.8-0.95, i.e. scattering occurs mostly in the forward direction. Although this is high, and the photons are generally travelling in a forward direction, for a fixed *µ_s_* there is still scatter at the focus. Photons will reach roughly the same location as with a *g* of 1, however the size of the focal spot is greater affecting image resolution.

In a scattering material the mean free path, $MFP=1/\mu_{s}$ , describes the average distance travelled by a photon between scattering events and the transport mean free path, $TMFP=1/\mu_{s}^{\prime }$ , describes the average distance travelled before the light becomes diffuse. In other words, the TMFP can be thought of as the average distance travelled by a beam before direction of individual photons have no relation to each other, making tasks like focusing or controlling the direction of a beam non-trivial.

The decay of ballistic photons is given by: $I_{out}=I_{in}e^{-(\mu_{a}+\mu_{s})l}$ [11]. As explained above the reduced scattering coefficient may be thought of as an effective scattering coefficient taking account the directionality of the scattering as represented by the g factor. For this reason, we measure of the decay of the light as:

(2) $$I_{out}=I_{in}e^{-(\mu_{a}+\mu_{s}^{\prime })l}$$

where the reduced scattering coefficient replaces the scattering coefficient. The penetration depth at this distance will somewhat longer than that associated with purely ballistic photons but accounts for the fact that the directionality affects the strength of the scattering. It should be pointed out the different expressions are sometimes given in the literature for the penetration depth [12–15]. These measures of penetration are obtained for a point source of excitation and will in fact vary with distance from the source. For this reason, we take a pragmatic measure of penetration depth that assumes a relatively broad source given by Eq. (2). It must be emphasised that all measures will depend on the shape of the source, but the values given below provide a good starting point for comparison.

The penetration depth of a material, *l*, is defined as the depth at which the intensity has reduced to 1/e, or ∼37%, of its original value due to scattering and/or absorption.

### 2.1 Impact of wavelength on scattering and absorption coefficients of skin chromophores

Scattering and absorption are both highly wavelength (λ) dependent making it important to consider changes across a broad spectrum. Rayleigh and Mie scattering are important when considering skin and biological tissue. Rayleigh scattering being relevant for constituents within the sample that are smaller than the wavelength of light, such as haemoglobin molecules which are approximately 5 nm in diameter, and Mie scattering for objects that are similar in size or larger than the wavelength of light, potentially including melanin molecules which have a diameter of up to 800 nm. In the Rayleigh regime the scattering intensity is proportional to λ^-4^, whereas in the Mie regime the relationship is more complex but is typically λ^-1^. In general, this leads to a much higher scattering coefficient at the shorter wavelengths and, overall, a scattering coefficient that changes relatively smoothly with wavelength. Absorption spectra are very different to scattering spectra and tend to be much more diverse with clear peaks, this is because absorption is directly linked to the electronic transitions present in the components that make up the sample. In the skin, chromophores such as melanin in the epidermis and haemoglobin in the dermis are the major absorbers of light and the absorption spectra will depend on the amount of these chromophores present [16].

**Fig. 3.**
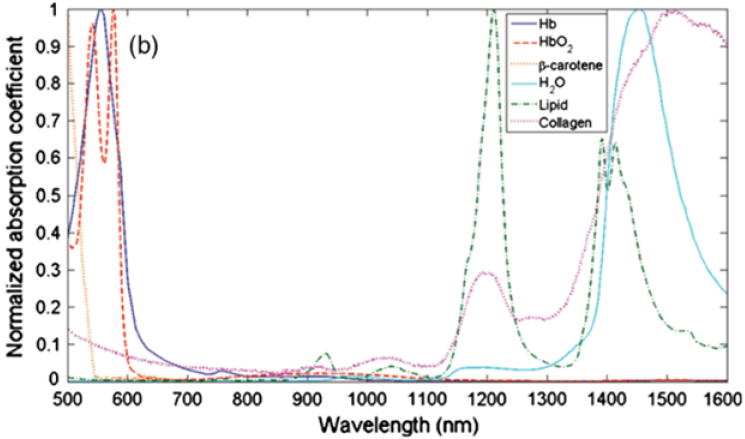
Absorption spectra in the visible and near-infrared region (500 to 1600 nm), normalised to their maximum, of oxygenated and deoxygenated haemoglobin, water, collagen, beta-carotene and lipid, the main constituents of the skin (Reprinted with permission from Ref. [18], Fig. 1(b))

Absorption makes imaging difficult; the number of photons is reduced and the signal of interest will eventually become swamped by noise. To maximise imaging depth, the wavelength of light should be selected to avoid the multiple absorption peaks of the components of the skin. For visible and near infra-red (NIR) wavelengths, scattering dominates over absorption in skin, being 100 - 1000 times stronger (and reduced scattering being 10 - 100 times stronger) [10]. At visible wavelengths, light is absorbed by melanin and haemoglobin; whereas structures, such as cell components and melanosomes of less than 300 µm, give rise to scatter [10]. Between 600 nm and 700 nm absorption decreases and scattering becomes dominated by collagen and elastin bundles in the dermis [16]. Melanosomes also effect scattering in the 600 nm to 700 nm range and, due to their high refractive index in comparison to their surroundings, their scattering coefficient is at least one order of magnitude higher than their absorption coefficient at 650 nm, even though melanin is a strong absorber [17]. As the wavelength of light increases from the visible towards the near infra-red, absorption by water, collagen and lipids become more prominent despite not being significant in the visible part of the spectrum (Fig. 3). At NIR wavelengths, scattering of photons in skin tends to decrease and these wavelengths are able to penetrate deepest into the skin. The optical windows for imaging, therefore, consist of a trade-off between minimal absorption and minimal scattering.

The golden window where absorption and scattering are low and, importantly, imaging depth is optimal is in the NIR spectral region. For commercial optical coherence tomography (OCT) imaging of the skin, light at a wavelength of 1300 nm is commonly used. At this wavelength, water absorption is low enough to acquire 500 µm image depth penetration in the dermis [19]. Four optical windows in the NIR have been described: I (650 nm - 950 nm), II (1100 nm -1350 nm), III (1600 nm - 1870nm) and IV (2100 nm - 2300 nm). NIR I has been well characterised for imaging human tissue [12,18,20–25], and NIR II, III and IV have been shown to offer increased penetration and optical transparency over NIR I in a range of *ex-vivo* and *in-vivo* tissue including normal and malignant breast and prostate tissue, human aorta, pig and mouse brain and chicken and rat tissue [18,26–28]. In the NIR region water has the greatest effect on absorption. However, using *ex-vivo* rat tissue Golovynskyi *et al*., showed that NIR III has the potential to be optimal for imaging skin due to reduced water absorption in this region [29]. The relationship between transmission and tissue thickness for rat brain tissue imaging was determined and optical window III was shown to provide greatest imaging depth due to reduced scattering and absorption in this wavelength range [30]. Similar has been shown to be true for other tissues, and NIR II and III have also been exploited for deep-tissue high-resolution optical imaging [27,31–36].

## 3. Composition of the skin

In general terms, human skin is made up of approximately 65% water and 9% lipid [37]. However, factors such as age and obesity affect the composition with a doubling of the lipid content being reported in the abdominal subcutaneous fat content with high levels of obesity [38]. As described previously, the skin consists of different layers and each of these layers have different optical properties. The epidermis is a stratified layer separated from the vascularised dermis by a basement membrane. Impenetrable, terminally-differentiated keratinocytes form the outermost epidermal layer, the stratum corneum, and keratinocytes proliferate and differentiate in the other layers of the epidermis [39]. The dermis mainly consists of extracellular matrix composed of collagen and elastin fibres embedded in glycosaminoglycans, proteoglycans, and water and contains blood vessels, nerve endings, hair follicles, and glands as well as multiple cells types [40]. Basal epidermal keratinocytes are anchored to the basement membrane by multi-protein complexes and the basement membrane is secured to the extra cellular matrix of the dermis by proteins including collagen [39]. The subcutaneous tissue is composed mainly of adipose tissue [40].

The absorption and scattering coefficients of the different layers of the skin can provide information regarding tissue composition. The major chromophores that contribute to absorption and scattering in the skin are haemoglobin (oxygenated and deoxygenated), melanin, water and lipid. In the visible spectrum, melanin and haemoglobin have most effect on absorption. Oxyhaemoglobin has peaks at 418 nm, 542 nm and 577 nm, while deoxyhaemoglobin has peak absorption at 430 nm and 555 nm [41,42]. Melanin absorption peaks at 335 nm but this reduces steadily with increasing wavelength. In the visible spectrum water absorption is negligible. In the NIR spectrum (750 nm – 1400 nm) oxyhaemoglobin absorption has maximal absorption at 900 nm and deoxyhaemoglobin absorption peaks at 960 nm. Lipid absorption, which greatly affect the subcutaneous skin layer, peaks at 900 nm, but it also has minor absorption peaks at 1040 nm, 1210 nm, 1400 nm, 1730nm and 1760nm [18,43]. Water has multiple peaks with increasing wavelength, with minor peaks at 740 nm and 835 nm and major peaks at 970 nm, 1180 nm, 1430 nm, 1650 nm, 1930nm and 1975nm [25,44]. Beyond the NIR, water also has an absorption peak at 3400 nm [44]. Collagen, predominantly found in the dermal skin layer, has absorption peaks at 1050 nm, 1200 nm, 1500 nm, and 1725nm [45].

**Fig. 4.**
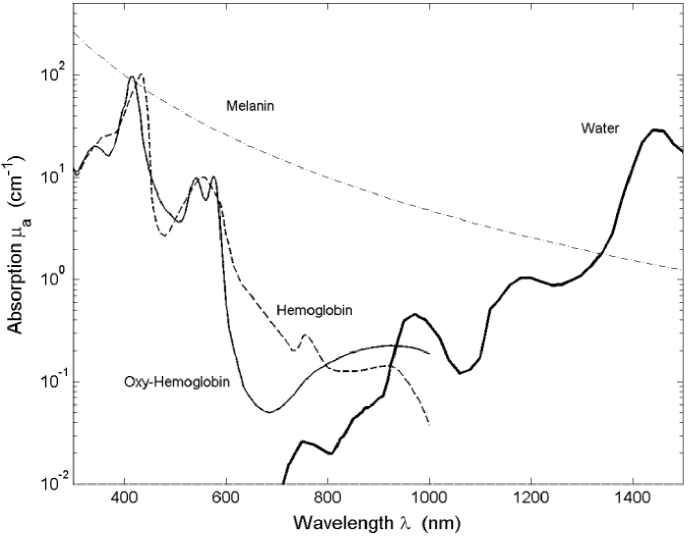
The absorption of skin chromophores in the wavelength range 300 nm -1500 nm with respect to their skin volume fraction (Reprinted with permission from Ref. [42], Fig. 2.4).

Although these chromophores have varying peak absorption across the visible to NIR spectrum, the amount they actually contribute to absorption in the skin is dependent on the amount or concentration of each in the skin. For example, although melanin absorbs strongly at visible wavelengths, it is only located in the very thin epidermal layer of the skin. Blood only makes up a few percent of the total skin volume, and is different depending upon the layer in question, therefore, although haemoglobin is a strong absorber, its total effect is modest [41]. Figure 4 shows the effect of chromophores on absorption when taking their fraction volume in the skin into consideration.

## 4. Determining the optical properties of the skin

**Fig. 5.**
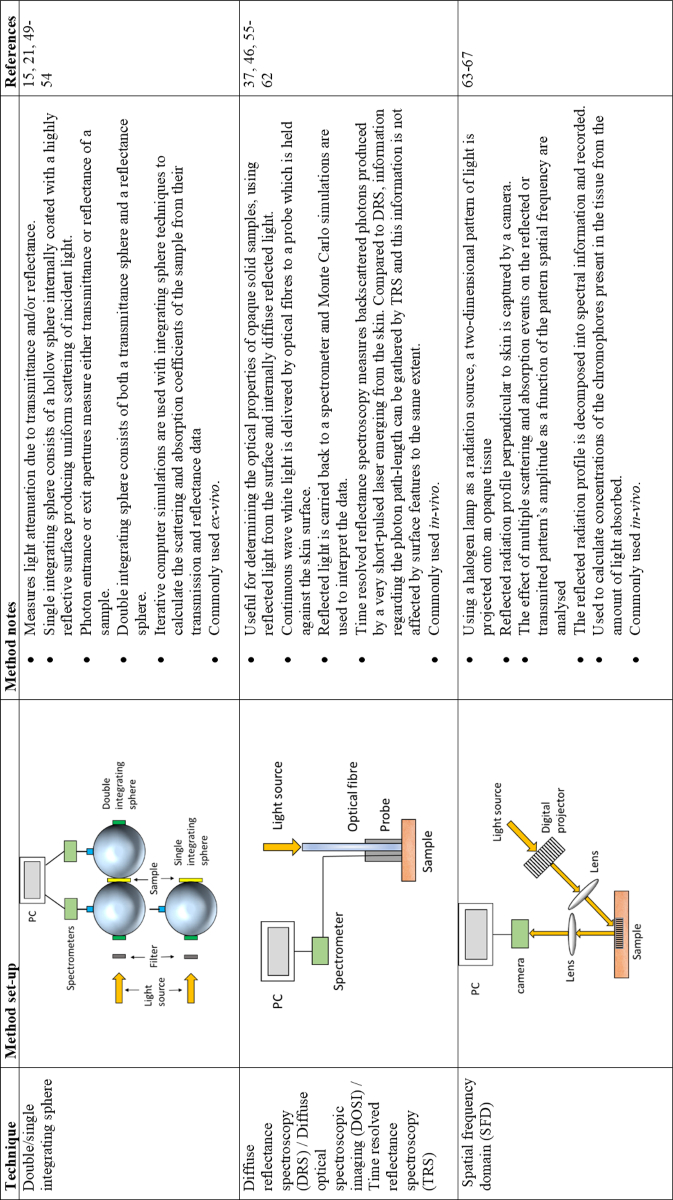
Methods used in the literature for determining the optical properties of the skin.

Methods used for determining the optical properties of skin both *ex-vivo* and *in-vitro* are limited. The main method used for taking measurements from *ex-vivo* samples have involved either one or two integrating spheres (Fig. 5). *In-vivo* methods generally involve spatial frequency domain (SFD) and diffuse reflectance spectroscopy (DRS; Fig. 5). Using *ex-vivo* samples, the optical properties of the separate skin layers (epidermis, dermis, and subcutaneous tissue in the simplest form) have been determined. Generally, the optical properties of individual skin layers cannot be directly measured for *in-vivo* samples; the data retrieved must be interpreted and modelled using, for example, diffusion models or Monte Carlo (MC) simulations, based on the known properties of the skin, including refractive index (1-35 -1.45 for biological tissues), diffuse reflectance and *g* from published information and thickness of the different skin layers [46,47].

### 4.1 Ex-vivo integrating sphere methods

Integrating sphere methods have commonly been used to determine the optical properties of *ex-vivo* samples. This method measures light attenuation due to amount of forward scattered light compared to the amount of backscattered light and collects and measures light over all angles [48]. Monem *et al*., showed comparative results for single and double integrating sphere optical property measurements of phantoms [48]. The calculated reduced scattering coefficients tend to become less similar with increasing sample thickness beyond approximately 2.4 mm. This potentially suggests that single and double integrating sphere methods are limited by sample thickness and this should therefore be a consideration when choosing a method for measuring the optical properties of samples.

Several authors have used integrating sphere techniques to determine the optical properties of the skin *ex-vivo* [15,21,49–54]. However, the data that they present all differ markedly, with 2–10-fold differences between absorption coefficients across a range of wavelengths and 2–3-fold variation in scattering coefficients.

Prahl obtained skin from the abdomen at autopsy, with the epidermis being separated following thermal treatment in a water bath [15]. The dermis was frozen and either cut with a cold dermatome or a freezing microtome to produce samples varying from 20 µm - 400 µm in thickness. The samples were bloodless; therefore, the effect of haemoglobin was not included in the measurement of absorption. Absorption and scattering coefficients were assessed using one sample for each thickness sandwiched between glass slides and a single measurement taken over a wavelength range of 450 nm - 800 nm. Prahl details some of the pitfalls in this measurement approach. During measurement, the thickness of the sample changed due to dehydration, therefore affecting the contribution of water to the coefficients being measured. A linear increase in anisotropy was shown to correspond with tissue dehydration and thinning which was proposed to reduce the distance between scatterers and increase their effective size. Prahl suggested that the optical depth was quite sensitive to (bloodless) tissue coagulation, which changes in the first 50 - 75 seconds of exposure to light [15].

To assess the effect of compression on the determination of optical properties of the skin, Chan *et al*., measured *ex-vivo* optical properties as a function of pressure at a spectral range of 400 nm - 1800nm using an integrating sphere with visible and IR detectors [49]. The skin from a Hispanic and two Caucasian donors was harvested from the buttocks and legs within 72 h post-mortem and contained epidermis and partial dermis. Optical properties for one sample from one donor (Caucasian female) across the spectral range were shown in graphical form in their paper. Absorption was shown to decrease rapidly from 1.4 mm^-1^ at 400 nm to 0.2 mm^-1^ at 1250 nm and to peak at 1450 nm to 2.1 mm^-1^, corresponding with maximal water absorption, with a sharp decrease in absorption thereafter. From 1700nm absorption starts to increase again with increasing wavelength. Scattering tended to decrease across the spectrum measured from 3.4 mm^-1^ to 0.6 mm^-1^. However, at 500 nm under varying pressure, even amongst samples from a single donor, the optical properties measured were variable, ranging from 14% difference between 3 absorbance measurements taken for an individual to 48%, and up to 95% difference between these measurements for scattering. With no applied pressure the absorption coefficient ranged from 0.34 mm^-1^ - 0.59 mm^-1^, with a 2-fold difference in measurements within samples from one donor. Under the same conditions, scattering coefficients varied from 2.13 mm^-1^ - 6.91 mm^-1^ amongst the donors. Overall, compression was shown to increase absorption and scattering coefficients by up to ∼75%, however it didn’t follow a monotonic trend.

Simpson *et al*., measured the optical properties of human abdominal and breast tissue within five days of harvesting from either plastic surgery or post-mortem examinations [50]. Samples were taken from five donors of varying skin pigmentation. Samples were refrigerated and returned to room temperature before being separated into layers consisting of epidermis and dermis, and 2 mm of subcutaneous tissue immediately below the dermis). Reflectance and transmittance of a spectrum of light ranging from 620 nm – 1000 nm through samples contained between glass coverslips and without compression were measured using a single integrating sphere. The optical properties for the epidermal and dermal skin section were 2.5 - 18.5-fold greater than those of the subcutaneous layer e.g., at 633 nm the mean absorption coefficient for the epidermal/dermal layer ranged from 0.033 mm^−1^ to 0.241 mm^−1^ compared to 0.013 mm^−1^ for the subcutaneous layer. Similarly, the reduced scattering coefficients at 633 nm ranged from 2.73 mm^−1^ to 3.21 mm^−1^ in the epidermal/dermal layers tested from 5 samples compared with 1.26 mm^−1^ in the dermis. Error in the data was large and attributed to differences in reflectance from imaging back to front and vice versa.

Using integrating sphere methods, Troy *et al*., measured the optical properties of the skin beyond the visible spectrum and into the NIR [54]. The aim of their work was to determine the mean optical properties of skin for a number of samples from 14 donors at multiple sites in the 1000 nm - 2200 nm wavelength range. Measurements were taken for samples consisting of the uppermost layer of the epidermis (the stratum corneum), the epidermis and the dermis. Variation in optical properties amongst the population was observed. The subcutaneous tissue was removed from each sample which were placed between glass slides, heated to 37 °C and measured from both sides within 24 hours of harvesting. An important assumption that Troy *et al*., made was the optical properties measured for *ex-vivo* skin samples are representative of *in-vivo* optical properties [54]. At 1000 nm absorption was recorded as an average of ∼ 0.1 mm^-1^, tending to increase slowly across the measured spectrum of 1000 nm - 2200 nm. Two absorption peaks were observed at 1460 nm and 1950nm with absorption being 2.2 mm^-1^ at 2050nm. This corroborates Simpson *et al*., whose data showed that absorption may increase beyond 1000 nm [50]. Between 1000 nm and 2200 nm scattering remained between 0.8 mm^-1^ and 1.3 mm^-1^ and tended to decrease with increasing wavelength.

Salomatina *et al*., collated data from freshly discarded specimens of normal and cancerous human skin obtained from surgeries in the spectral range 370 nm - 1600 nm [52]. Skin samples were analysed within 7 h of surgery, separated into the three skin layers, sectioned with a micro-cryotome into slices of varying thickness and hydrated with saline before being sealed between two coverslips. Reduced scattering coefficients decreased with the increasing wavelength in all skin layers. An increase of reduced scattering coefficient at 1450 nm in the region of strong water absorption was noted. Scattering in the epidermis was shown to be higher than in the remaining layers across the spectrum tested. In the epidermis, scattering decreased from ∼11 mm^-1^ at ∼360 nm to 2.3 mm^-1^ at 1600 nm. Dermal and subcutaneous tissue scattering coefficients were similar across the spectrum measured, ranging from ∼4.5 mm^-1^ and ∼3.5 mm^-1^ at ∼500 nm to 1.7 mm^-1^ and ∼1.5 mm^-1^ at 1600 nm respectively. Although the epidermis in these samples contains small amounts of melanin, the increased scattering compared to the dermis and subcutaneous tissue layers can be explained by the difference in refractive index of melanin compared to its surroundings.

Similarly, these authors described absorption in the epidermis being greater than in the dermis and subcutaneous tissue and that this decreased with wavelength except for a peak at 1450 nm corresponding with water absorption. Minimal absorption was observed at 1100 nm for each skin layer. Absorption in the epidermis, dermis and subcutaneous layer ranged from ∼1.5 mm^-1^, ∼1 mm^-1^ and ∼1.8 mm^-1^ at ∼400 nm to 0.8 mm^-1^, ∼0.8 mm^-1^ and ∼0.4 mm^-1^ at 1600 nm respectively; on average a reduction of ∼2-fold for the absorption coefficient at 1600 nm compared to 400 nm.

The optical properties of Asian skin have been measured *ex-vivo* at 400 nm - 1100 nm by Shimojo *et al*., using a double integrating sphere spectrometric system and a white light source, and compared to Caucasian and African skin data reported by Simpson *et al*., and Salomatina *et al.* [50,52,53]. The aim of this work was to determine appropriate irradiation protocols for laser therapy and diagnosis. Like Salomatina *et al*., skin samples were taken from various locations across the body and separated into three skin layers with epidermal and dermal layers being analysed within 50 hours of collection and subcutaneous tissue within 12 hours. The layers were stored at 4 °C until they were sectioned and placed between glass slides with minimal compression for analysis. Peak absorption and scattering coefficients for all skin layers was at 405 nm steadily reducing with increasing wavelength with the greatest absorption and scattering coefficients being in the epidermis. At the wavelength spectrum of 405 nm - 1064 nm, absorption ranged from 3.32 mm^-1^ - 0.13 mm^-1^ and reduced scattering ranged from 9.95 mm^-1^ - 2.85 mm^-1^ in the epidermis (standard deviations for both coefficients tended to reduce with increasing wavelength). Shimojo *et al*., also considered the depth to which light penetrates into the skin at different wavelengths; at 405 - 532 nm, the light penetrates to a maximum depth of ∼0.3 mm reaching the upper part of dermis [53]. Beyond 980 nm, light has the potential to penetrate the subcutaneous tissue travelling a distance of ∼1.65 mm.

### 4.2 In-vivo measurement of optical properties of the skin

Measuring the optical properties of skin non-invasively *in-vivo* makes determining the optical properties of the different skin layers difficult. Many methods for determining the optical properties of skin *in-vivo* have been described by multiple authors. Diffuse reflectance spectroscopy (DRS) has been used by several authors and uses reflected light from the surface and diffuse reflected light from within the sample [46,55–61]. The concentrations of tissue chromophores including melanin, haemoglobin water and lipid can also be determined using DRS measurements [56]. Torricelli *et al*., used time resolved reflectance spectroscopy (TRS), to measure backscattered photons produced by a very short-pulsed laser emerging from the skin [37,62]. Spatial frequency domain spectroscopy (SFD), that consists of projecting a two-dimensional pattern of light onto an opaque tissue, is also commonly used *in-vivo* [63–67]. An overview of these three techniques can be found in Fig. 5. Like *ex-vivo* data, *in-vivo* data are again variable, and these will be discussed further in the following.

#### 4.2.1 In-vivo measurement using diffuse reflectance spectroscopy (DRS)

Taking measurements from 18 subjects in the wavelength range 500 nm-1000 nm, Tseng *et al*., showed that there was a 2-fold decrease in absorption as the wavelength increased from 500 nm to 600 nm [55]. In the visible region both melanin in the epidermis and haemoglobin in the blood supply to the dermis contributed to the high absorption coefficients in some subjects. A small peak in absorption was observed at ∼970 nm for all skin-types corresponding with water absorption. These authors showed that skin location and colour have important effects on measured absorption and scattering in the skin. MC simulations were used to determine the penetration depth of photons in the skin and showed that the interrogation depth at 500 nm was at least 46% less than at 900 nm. Because of the change of penetration depth associated with wavelength, it was suggested that different wavelengths could be used to determine the optical properties of different skin layers.

DRS has been used to determine scar severity to understand the therapeutic response of the scar tissue [56]. At 800 nm the absorption coefficients described by Tseng *et al*., were 5-fold greater than those measured by Hsu *et al.* [55,56]. However, their scattering and absorption profiles followed a similar pattern to the other authors’ with absorption being greatest between 500 nm and 600 nm beyond which it declines steadily with increasing wavelength except for a small peak observed at ∼970 nm. Like Tseng *et al*., skin location was shown to have an important effect on absorption and scattering coefficients by Doornbos *et al.* [55,57]. Wavelength measurements were limited to 630 nm, 660 nm and 700 nm and at 630 nm absorption was greatest in the forehead compared to the sole of the foot and the arm. At 660 nm it was greatest in the sole of the foot, and at 700 nm the arm had greatest absorption. However, scattering was greatest in the forehead and least in the arm at each of these wavelengths. Numerous authors have measured the optical properties of skin on the forearm using DRS and the absorption coefficients vary 9-fold amongst the publications from 0.01 mm^-1^ - 0.09 mm^-1^ at 700 nm [37,46,55,57,60]. Similarly, measured reduced scattering coefficients have up to 10-fold variability using DRS at this wavelength, ranging from 0.184 mm^-1^ to 2.19 mm^-1^ [55,57,60,61].

Using time-resolved reflectance spectroscopy (TRS), Torricelli *et al*., evaluated of the optical properties of skin from different regions of the body (arm, abdomen, and forehead) *in-vivo* from 610 nm to 1010 nm [37]. Again, scattering decreased progressively with increasing wavelength, while the absorption showed the features of absorption by water, haemoglobin, and lipid, with average spectral peaks at ∼600 nm, 760 nm and 950 nm of approximately 0.017 mm^-1^, 0.013 mm^-1^ and 0.027 mm^-1^ respectively. The amount of absorption associated with each of these skin constituents depended upon skin location and the most absorbing locations varied amongst subjects. In agreement with Doornbos *et al*., the forehead was the location of greatest scattering amongst the subjects and the arm the least [57]. This may have been due to 1 cm - 2 cm tissue depth being probed by photons, the skin being thinnest at the forehead and photons being scattered by the skull beneath. Salomatina *et al.*, suggested that scattering properties were dependent upon location and that collagen-elastin networks in fat from the scalp were thicker and denser than those in the subcutaneous adipose tissue of skin taken from the back [52]. This resulted in increased scattering coefficients compared to the back where connective tissue was thin.

DRS has also been used by Bosschaart *et al*., for diagnostic and therapeutic procedures in neonates and realistic determination of the optical properties of the skin is important for improvement of current treatment regimens [59]. The values of absorption and scattering coefficients measured for the skin can be used to determine the distribution and transmission of light in the skin, thereby improving understanding for optical diagnostic/therapeutic probe design.

Hung *et al*., is one of few authors to use DRS to take optical measurements of the skin beyond 1000 nm, taking measurements between 650 nm - 1350 nm [60]. Their data showed a downward trend in optical scattering across the spectrum from 2.45 mm^-1^ to 1.15 mm^-1^; however, the standard deviations for measurements taken above 1000 nm are large suggesting no significant differences between the scattering coefficients beyond 1000 nm. Peaks in absorbance were observed at 970 nm and 1150 nm - 1200 nm corresponding with water absorption. Between 1200 nm and ∼1275 nm the absorbance declined rapidly and then increased sharply beyond this wavelength.

Using DRS, Jonasson *et al*., measured optical scattering properties *in-vivo* for the largest cohort of the publications reviewed; 1734 subjects [61]. The reduced scattering coefficient decreased in the spectrum of 475 nm - 850 nm from 3.16 mm^−1^ to 1.13 mm^−1^. They processed this data based on gender and age; there was a significant difference between men and women; measured reduced scattering coefficients were greater in men than women across the spectrum measured. As age increased the reduced scattering coefficient decreased, potentially due to decreasing collagen concentrations in skin with age.

#### 4.2.2 In-vivo measurement using spatial frequency domain (SFD) methods

The absorption and scattering coefficients of skin from 198 Asian subjects based on age and skin location (inner forearm, cheek, dorsal surface of hand, and between thumb and forefinger of the hand) were determined using SFD in the 400 nm - 1600 nm spectral range by Kono *et al.* [63]. Across the subjects the average absorption coefficients amongst all data showed spectral peaks at 600 nm, 1000 nm, 1200 nm and 1450 nm corresponding with absorbers in the skin, with main differences between locations being observed in the visible spectrum. Scattering ranged from 12 mm^-1^ down to 3.7 mm^-1^ between 450 nm and 1400 nm.

Saager *et al*., measured the optical properties of the dorsal forearm for 12 subjects [64]. Although their data followed the same general trends as that of other authors, the optical properties were only measured up to 900 nm so peaks associated with the water beyond this were not observed. At 900 nm both the absorbance and reduced scattering in the skin of all subjects was similar, however at shorter wavelengths variability between measurements was greater. Both reduced scattering and absorption tended to decrease with increasing wavelength. These authors showed that there were clear differences between both measured absorption and reduced scattering coefficients when subjects were grouped based upon their skin pigmentation.

Phan *et al*., determined the reduced scattering and absorption coefficients of skin between 471 nm and 851 nm from 15 subjects at varying locations and with diverse skin pigmentation [65]. They found that the least variation in scattering between subjects was at 851 nm. Baseline scattering varied amongst measurement location. There were not enough measurements made across the spectrum to observe absorption peaks associated with the components of the skin, although the general trend was for a reduction in absorption with increasing wavelength. Like Saager *et al*., these authors showed that skin pigmentation affected the measured coefficients and that inter-subject variation for both absorption and scattering was found to decrease amongst all locations measured with increasing wavelength [64,65]. This may be attributed to melanin absorption being lowest at 851 nm (the longest wavelength measured by these authors), which not only affects absorption measurements at this wavelength but also reduced scattering measurements due to the ability of photons to penetrate deeper, and therefore to be scattered by skin components at greater depths.

SFD imaging, discussed here, is limited due to slow and sequential acquisitions of light patterns and edge artifacts and is therefore difficult to apply to clinical applications that require real-time feedback such as surgery [68]. Improvements to SFD imaging have been made by Aguénounon *et al.,* to enable its use in surgical settings, by using Single Snapshot imaging of Optical Properties (SSOP). This reduces the number of image acquisitions to one and applies machine learning approaches to improve accuracy, reduce edge artifacts, improve resolution and increase imaging speed. This could potentially be further improved through the use of a new SFD model that reduces error amongst the extracted tissue optical properties using SFD imaging and incorporates phase function information [69]. This model reduced the median relative error by 10% for µ_s’_ and 64% for µ_a_ compared to previously used models.

When comparing the two most commonly used methods for determining the optical properties of the skin *in-vivo* amongst the published data, the reduced scattering coefficients tend to be larger when measured using DRS than SFD being from 1.2 - 1.6-fold greater in the 500 nm - 100 nm spectrum. Conversely, amongst the published data SFD absorption measurements tended to be between 1.3 - 1.8-fold greater than DRS in the 500 nm - 700 nm spectrum and at 1000 nm. However, at 800 nm there was little difference between the two methods.

## 5. Differences amongst the average reduced scattering and absorption coefficients published for ex-vivo and in-vivo data

**Fig. 6.**
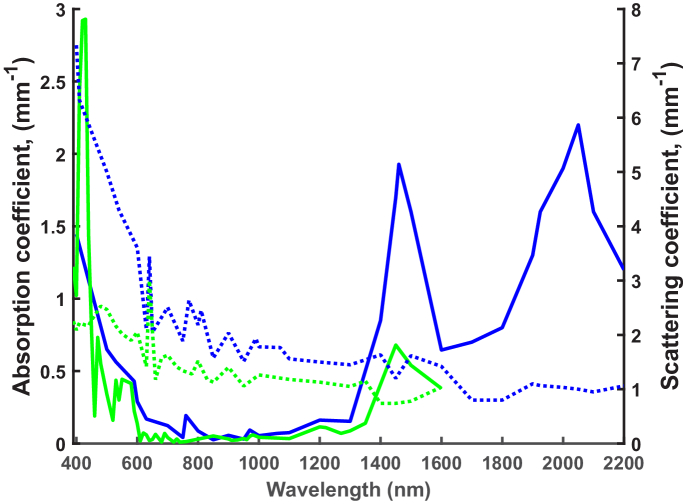
Comparison of average absorption and reduced scattering coefficients collected from the published *ex-vivo* (blue lines) and *in-vivo* (green lines) data. Scattering coefficients are shown as dashed lines and absorption coefficients as solid lines.

The mean absorption and reduced scattering data were collected from nine *ex-vivo* publications and twelve *in-vivo* publications after an extensive literature search and plotted as shown in Fig. 6. In general, the *ex-vivo* and *in-vivo* absorption and reduced scattering show similar trends. However, the average *in-vivo* reduced scattering values beyond 480 nm are approximately 1.7-fold less than in the *ex-vivo* situation. In the 800 nm -1300 nm spectral range this difference in reduced scattering coefficients decreases to 1.4-fold. For both data sets, a large scattering peak is observed at 640 nm. This is the most significant peak amongst the *in-vivo* data. This does not tend to be seen in the published data when taken individually; reduced scattering coefficients generally reduce linearly with increasing wavelength. However, a significant peak was observed at 640 nm for the *in-vivo* data published by Hsu *et al*., potentially representing scattering due to collagen bundles in the skin [56]. By averaging the *ex-vivo* published data, this peak became more apparent. Both *in-vivo* and *ex-vivo* absorption and reduced scattering data display multiple peaks, some being true resonant peaks whilst other represent the noise amongst the data.

The wavelength range for *ex-vivo* data was greater than for *in-vivo* data, ranging from 400 nm - 2200 nm and 390 nm - 1600 nm respectively. Therefore, comparisons cannot be drawn beyond 1600 nm between these data. Minimum absorption for both *ex-vivo* and *in-vivo* data is between 800 - 1300 nm. In this spectral range the *ex-*vivo absorption coefficients are approximately 1.6-fold greater than *in-vivo* absorption coefficients (compared with a 2.5-fold average difference across the whole spectrum). Major absorption was observed between 400 nm - 600 nm for both *ex-vivo* and *in-vivo* data sets due to melanin absorption, however the absorption peaks in this region are more distinct for the *in-vivo* data. Absorption tends to increase for both data sets beyond 1300 nm. In general, the wavelengths of absorption peaks correspond with scattering troughs, which may be because high absorption makes scattering difficult to measure.

A small peak in absorption at 760 nm and major absorption peaks at 1460 nm and 2050nm, representing major water absorption peaks are observed for *ex-vivo* data. *In-vivo* a water absorption peak at 1450 nm similar to that observed at 1460 nm for *ex-vivo* data is apparent, although this is 2.5-fold less than for *ex-vivo.* These peaks may be greater in the *ex-vivo* data due to sample preparation techniques – ex-vivo samples are often hydrated in saline and their water content is not representative of the *in-vivo* situation. There is a corresponding trough in scattering at 1450 nm for both datasets. This may be an artefact due to high absorption at this wavelength leading to a reduction in the photons available to be scattered, hence a reduction in the number detected and an underestimation of the scattering coefficient. Beyond 1450 nm *in-vivo* reduced scattering increases which has the potential to affect deep imaging and needs to be investigated further. It is difficult to draw conclusions from this as *in-vivo* data has not been published beyond 1600 nm. The measurement spectrum for the published *ex-vivo* dataset extends beyond that of the *in-vivo* dataset and, although the scattering coefficient increases between 1460 nm - 1500 nm, it steadily decreases beyond 1500 nm. It would be useful to determine the scattering and absorption coefficients in this region of the spectrum to determine whether *in-vivo* measurements follow a similar pattern to *ex-vivo* data between 1600 nm - 1800nm, where scattering and absorption troughs are coincident. The 1600 nm - 1800nm range corresponds to window NIR III, if the scattering and absorption coefficients of skin are shown to dip here this could prove to be an important target for medical imaging optically.

Scattering in skin is the main loss mechanism of photons dominating over absorption. *In-vivo* scattering coefficients range from approximately 1–150-fold greater in value than absorption coefficients across the spectrum, whilst *ex-vivo* reduced scattering coefficients range from 1–60-fold greater than corresponding absorption coefficients. Hence, for both *in-vivo* and *ex-vivo* cases a reliable dataset providing optical properties of the skin across a broad light spectrum is required.

## 6. Factors affecting absorption and scattering and reasons for sample variation amongst the published ex-vivo and in-vivo data

Table 1 summarises the absorption and scattering properties of the skin, taken from the literature, at wavelengths corresponding to the significant absorption peaks of the major chromophores in human skin. The data have been split into different Fitzpatrick skin-type groups which classifies skin based on colour and its response to UV with Fitzpatrick skin-type I being the palest skin and skin-type VI being the darkest. At wavelengths in the visible spectrum, absorption by the epidermis is dominated by melanin which decreases as wavelength increases. Absorption in the dermis and subcutaneous tissue is dominated by the presence of blood and haemoglobin. As the wavelength of light increases into the NIR absorption by all skin layers becomes dominated by water and lipid content. Both inter- and intra- sample variation is observed amongst these data and there are many holes in the data (standard deviations for individual data from publications are not shown in the table because in many cases they are not published). The reasons for this variation are going to be discussed in this section.

**Table 1.** Absorption and scattering coefficients (mm^-1^) measured by various authors at wavelengths corresponding to the skin constituent chromophore spectral peaks. E and E + D represent data taken from the epidermis and epidermis plus dermis respectively; Data from Ref. [65] is at wavelengths shown in the superscript; FST = Fitzpatrick skin-type; *published scattering data converted to reduced scattering where g = 0.84 and is equal to the average g presented in the published data.

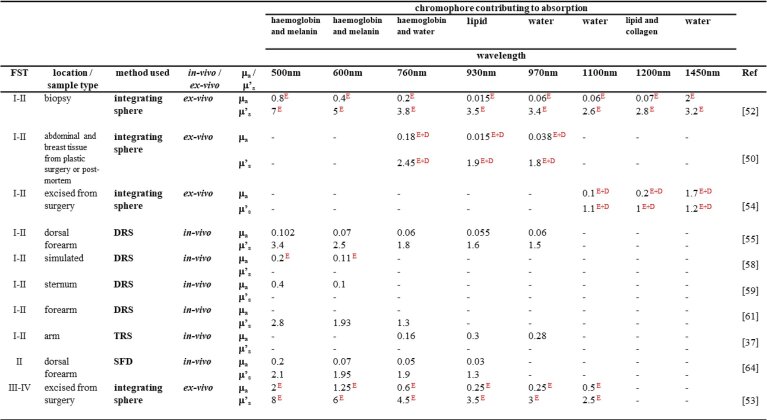

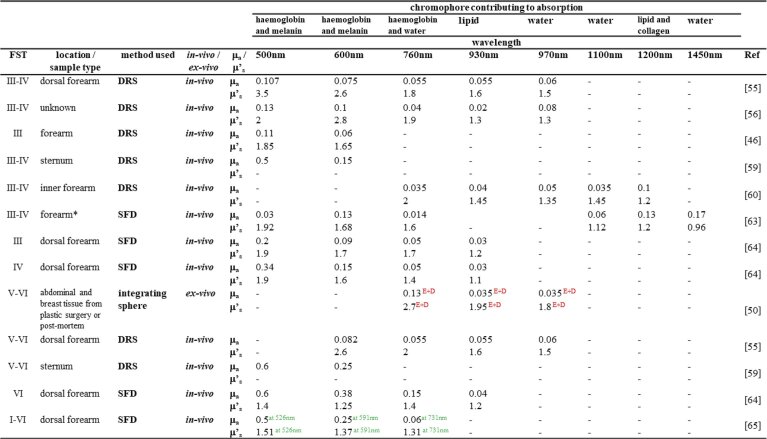

**Fig. 7.**
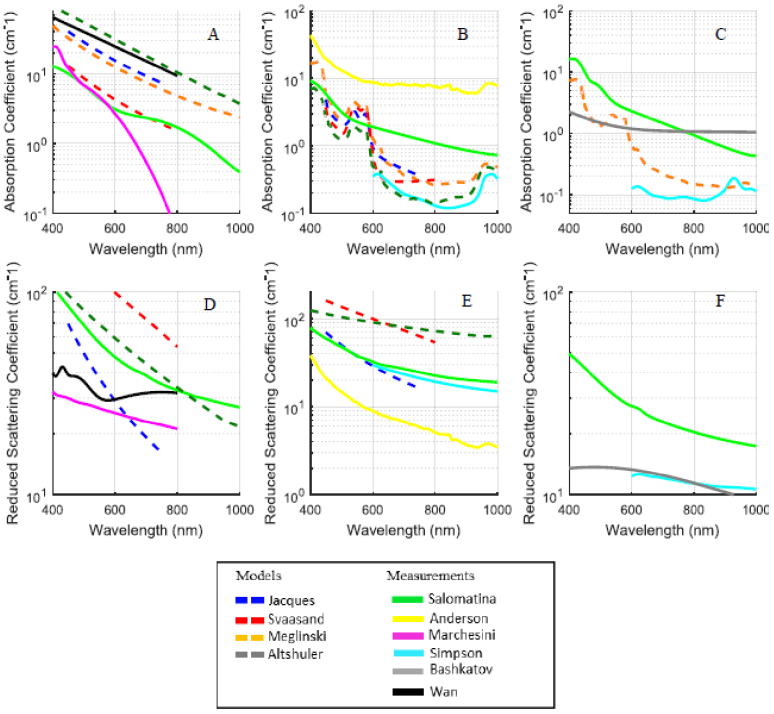
The variability of reported data. Ex-vivo absorption and scattering coefficients versus wavelength from published data for epidermis (A, D), dermis (B, E) and subcutaneous tissue (C, F). Note that units for the coefficients are cm^-1^. Solid lines represent data extracted from the experimental measurements; dashed lines represent data from the mathematical models. (Reprinted with permission from Ref. [8], Fig. 1 © The Optical Society).

As discussed, many authors have measured the optical properties of skin *ex-vivo* (section 4.1). However, there are several alterations that occur in *ex-vivo* skin compared to *in-vivo* that need to be considered when establishing the reliability of the optical properties determined. Firstly, there is a potential difference between samples taken as biopsies from live subjects compared to samples taken post-mortem. Post-mortem, skin takes on a blue/purple discolouration due to pooling of blood, and clotted blood within the sample may become difficult to remove from the sample and interfere with measurements. Generally, biopsied samples are exsanguinated, so no blood is present. The time to measurement post-harvest varies amongst authors from hours to a matter of days. Time will influence decomposition of the cells within the sample and potentially, therefore, the optical properties being determined. *Ex-vivo* samples were treated differently amongst the publications prior to optical measurement including exposure to heat to remove the epidermis, freezing for storage and slicing, various slice thickness and dehydration associated with this, rehydration of samples in saline, averaging measurements taken of the samples from front-to-back and back-to-front, and potential unknown compression of the samples between glass slides. In part, due to inconsistencies with the sample treatment, the published data is inconsistent. This, in-turn, affects the results that can be retrieved from MC simulations leading to variability in the predicted photon density at given depths of the skin and therefore significantly affects our ability to understand how deeply into the skin we could potentially image. This is also an issue when these values are used to select light treatment or diagnostic options and Mignon *et al*., who reviewed *ex-vivo* data sets, questioned the validity of the reported values (see Fig. 7) [8]. They showed that the modelled data predicted absorption at the expected spectral peaks for skin, where this was not present in the measured *ex-vivo* data (for example, Fig. 7(b) and (c) show that peaks corresponding to haemoglobin absorption were not present in measured *ex-vivo* data, probably due to sample treatment). Note that the data shown in Fig. 7 has coefficient units of cm^-1^ unlike the remainder of the paper where the units used are mm^-1^.

Figure 7 also shows the variability amongst the published *ex-*vivo data. Reviewers of *ex-*vivo data all agree that the variability of the reported data is a problem and the comparability of ex-vivo data with the in-vivo optical properties is also questioned [8,12,23–25,62]. Therefore, the published *in-vivo* data may be of more use for determining the behaviour of light in the skin and therapeutic applications. However, *in-vivo* measurements are not immune to variation. Following on from the work by Mignon *et al*., we have investigated the variability of the optical properties of human skin amongst the published *in-vivo* data [8]. Wide inter- and intra- publication variability has been shown. The wavelength ranges, measurement site, methods and pressure applied when taking measurements, photo-exposure of the measurement site and skin pigmentation, and sample size for the published data was diverse. This makes comparing and interpreting the published data difficult; however, broad conclusions may be drawn. The variation in reported *in-vivo* data is often exacerbated by publications that do not report on the measurement site nor skin pigmentation of individual subjects [56,58,59,65]. The published data gathered and analysed here show that variation amongst the *ex-vivo* data is far greater than the *in-vivo* data having up to 77-fold differences amongst the absorption data compared with up to 9.6-fold differences respectively.

**Fig. 8.**
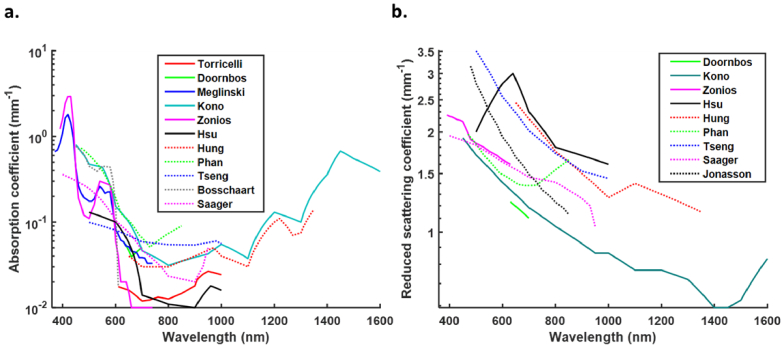
Variability in absorption coefficients (a) and reduced scattering coefficients (b) amongst the published *in-vivo* data. For readability the data is plotted by linear interpolation of the available data points. Note that Kono *et al.,* measured scattering coefficients which were converted to reduced scattering coefficients for the purpose of this graph using the equation µ’_s_ = µ_s_(1-g) and g = 0.84 (the average anisotropy coefficient amongst all the published papers used in this review).

The graphs in Fig. 8 show the variability amongst the absorptions and reduced scattering coefficients for the published *in-vivo* data. Trends can be observed amongst the published data, similar to those shown by Mignon *et al*., for *ex-vivo* data (Fig. 6), but an understanding of the most useful data set for modelling transmission of light through the skin is difficult to ascertain [8]. Generally, there is a sharp decline in absorption coefficient between ∼420 nm and 460 nm, however only three of the published datasets cover this spectrum [46,58,64] (Fig. 8(a)). A small peak is observed amongst some of the published data at around 550 nm - 580 nm corresponding with melanin absorption and absorption coefficients tend to become minimal in the 700 nm - 900 nm spectrum [46,58,59,63]. However, this is untrue for data published by Tseng *et al*., and Doornboss *et al*., whose data tend to show an increase in absorption coefficient between 630 nm - 700 nm and 730 nm - 850 nm respectively [55,57]. Beyond 900 nm there is a tendency for absorption to increase, however few of the published data sets measure absorption beyond 1000 nm. Measurements published by Kono *et al*., and Hung *et al*., beyond 1000 nm show similar absorption peaks and troughs [60,63]. However, measurements taken in the 1000 nm - 1350 nm by Hung *et al*., are up to 2-fold greater than those measured by Kono *et al*. This is potentially of importance for applications that might use longer wavelengths equivalent to the higher NIR windows. Note that Kono *et al*., collected measurements from 198 subjects compared to only one by Hung *et al*. These graphs show that the data are very different in terms of sample number and measurement spectrum and, although their trends corroborate each other within minimal spectra, it would be difficult to reliably choose a useful data set.

Reduced scattering data also display inter-publication variability amongst the *in-vivo* data (Fig. 8(b)). The trend is for decreasing reduced scattering coefficients with increasing wavelength, however, between 1000 nm and 1100 nm there is a small increase in the reduced scattering coefficients published by Hung *et al.* [60]. Further data collection is required to draw any conclusions about trends at longer wavelengths.

As reported for *ex-vivo* data there are multiple potential reasons for the variability observed in the reported data including instrument pressure, skin measurement location, external temperature, subject gender, age, obesity and skin pigmentation some of which will be discussed briefly.

Instrument pressure when applied to the skin surface can affect the optical properties being determined. Application pressure is not generally well controlled for and can cause deformation of the tissue being measured and distort the optical properties being measured [70]. The effect of pressure when taking optical measurements of the earlobe using DRS was assessed by Li *et al.* [71]. They showed that increasing pressure increased diffuse transmittance and the calculated reduced scattering coefficients particularly at longer wavelengths. As well as reducing sample thickness, increasing pressure is likely to displace free water in the skin and affect the measurement of haemoglobin, both of which will have consequences on the resulting optical coefficients measured [70,72]. Optimal contact pressure for DRS measurements was determined to be between 10 kPa – 25 kPa [71].

Location has a major effect on optical properties since the thickness of the skin layers is variable at different sites. For example, mean epidermal thickness in samples from 71 subjects has been shown to range from 74.9 µm to 96.5 µm on the dorsal forearm and buttock respectively [73]. Oltulu *et al*., measured epidermal and dermal thickness in 180 male and female volunteers across 6 different body locations (scalp, abdomen, back, top of foot, top of hand, and the breast) and found a greater range of epidermal thickness [74]. The thickest epidermis was found on the top of the foot (267.4 µm) and the thinnest on the breast (76.9 µm) in women, while mean male epidermal thickness ranged from 244.8 µm to 112.4 µm on the top of the hand and the scalp respectively. Sample preparation methods may have contributed to the broad range of thickness amongst the epidermal measurements between these two studies. Oltulu *et al*., also made dermal thickness measurements and found that mean dermal thickness in females was greatest on the breast and least on the top of the hand (4717.1 µm to 2115 µm respectively) [74]. However, for males mean dermal thickness ranged from 2363 µm to 5888 µm on the top surface of the foot and the breast respectively. However, intra-site variability of measured thickness was also shown to be large.

Temperature has been shown to have an important effect on the optical properties determined in the skin. *Ex-vivo*, Laufer *et al*., showed that increasing temperature from 25 °C – 40 °C produced changes in reduced scattering coefficients of the layers of the skin, with an increase in the dermis, but a decrease in the subcutaneous tissue measured over the 650 nm –1000 nm spectrum [75]. Their work showed no significant changes in the absorption coefficients with increasing temperatures. Iorizzo *et al*., determined the optical properties of mouse ear skin *in-vivo* at temperatures ranging from 36 °C – 60 °C and wavelengths ranging from 400 nm-1650 nm [76]. By fitting to simulated MC and matching diffuse reflectance and transmission of skin samples to deduce the optical properties from measurements taken, they showed that absorption coefficients increased with temperature, while anisotropy coefficients decreased. Between 400 nm – 950 nm reduced scattering increased with temperature; beyond this, it decreased with temperature compared to measurements taken at lower temperatures.

Kono *et al*., showed that gender and age affected measured optical properties [63]. Contrary to results shown by Jonassen *et al*., females of all age groups possessed greater reduced scattering coefficients than males of all age groups across the wavelength range measured (450 nm – 1600 nm) [61]. Differences in these coefficients with age and gender may be attributed to differences in collagen and elastin content as skin ages, and natural differences in collagen and adipose tissue content for males and females [77]. Males tend to have greater skin collagen content than females, but less adipose tissue. Similarly to Jonassen *et al*., these authors showed that scattering decreased with age regardless of gender within the spectrum measured. However, absorption in males of all age groups was greater than for females in the visible spectrum and these coefficients increase with age. This is difficult to explain, but in general male skin is thicker than female skin and age-related changes may be due to differences in oestrogens and androgens in males and females which affect epidermal and dermal thickness [78]. Beyond 1150 nm, absorption is similar for both male and female subjects regardless of age, tending to increase with increasing wavelength; absorption peaks in the NIR were observed at approximately 1000 nm, 1200 nm and 1450 nm with the absorption coefficient at 1450 nm being equivalent to that at 450 nm. This decrease in measured reduced scattering and increase in absorption coefficients could be explained by Kourbaj *et al*., who showed that there was a correlation between increasing dermal water and decreasing collagen content with increasing age [79].

Finally, but equally importantly, skin pigmentation affects the optical properties of the skin particularly in the visible spectrum where melanin absorption is dominant. Due to high concentrations of melanin in the epidermis of dark skin, strong absorption limits penetration by light in the visible spectrum affecting the scattering signal that can be detected at depth. Therefore, at visible wavelengths, scattering coefficients measured are linked to the scattering properties of the epidermis [64]. At NIR wavelengths, melanin absorption decreases, penetration depth can increase, and the scattering vales recorded become mainly affected by the dermal layer of the skin. Tseng *et al*., suggested that the relative decrease in scattering associated with increasing melanin in the skin, and discussed by several authors, may be due to fewer photons reaching the dermis, the layer that would be expected to contribute strongly to scattering in the NIR [55,61,64,65]. Additionally, photo-exposure at different sites also varies, for example, the dorsal forearm will likely have been exposed to more sunlight than the upper inner arm. This causes varying levels of melanin on an individual basis, which in turn affects absorption, particularly in the visible spectrum.

Mignon *et al*., discussed the challenges relating to the published data being that individual references did not provide sufficient information since not all relevant optical properties for all skin layers have been measured over a wide optical range [8]. Therefore, data must be combined from different publications. Due to the variability in the data amongst different publications for the reasons already discussed, combining data presents its own issues. To achieve consistency and gain a detailed understanding of the optical properties of skin, there is a real need for a dataset providing optical properties over a range of wavelengths for a variety of skin pigmentations, locations, genders, and ages. However, trends can be extracted from the published data.

*In-vivo* data should be used for modelling the propagation of light through the skin because the blood and water content of the tissue remains realistic; treatment of tissue *ex-vivo*, such as time to measurement, storage conditions, compression, exsanguination, and hydration all produce changes in the optical properties and with unrealistic absorption coefficients being determined. *In-vivo* coefficients have been found to be an order of magnitude lower than those determined for *ex-vivo* samples and this is particularly important at wavelengths greater than 1000 nm where water content affects the optical properties of the skin [50,60]. The major contributors to variability amongst *ex-vivo* and *in-vivo* data are likely to be methods used, measurement site and skin pigmentation. However, *ex-vivo* data has the additional complication of sample treatment methods and this is likely to be the main contributor to the inter-publication differences in the determined coefficients. It is important to note that, unlike *ex-vivo* sampling, for *in-*vivo measurements it is difficult to determine the volume of the skin being probed.

## 7. Photon penetration depth from published modelled data

Light penetration is an important factor for diagnostic imaging; the further light can penetrate and return for detection the deeper we can image [12]. An understanding of the optical absorption and scattering properties of tissues provides useful information regarding likely penetration depths. Recently, Finlayson *et al*., looked at simulated light propagation through a six-layered skin model in the 200 nm -1000 nm spectrum using MC [80]. They varied incident angles, stratum corneum thickness and compared direct and diffuse light sources and the effect of these on the lateral spread of light in skin. Penetration depth, which corresponds to the depth at which light intensity has reduced to 1/e (∼37%) of its original value, varied with wavelength and in relation to optical properties of the skin layers. Their models showed that for a direct light source approximately 1% of the light reached a depth of 5 mm in the wavelength ranges 625 nm -725 nm and 760 nm – 925 nm (covering the wavelength range for optical window NIR I). In general, it was shown that penetration depth increased with increasing wavelength up to approximately 725 nm, beyond which the penetration depth reduced. A small increase in penetration depth was associated with light at approximately 810 nm. As expected, diffuse light sources simulated by randomly choosing the incident angle of the light were shown not to penetrate as deeply as direct sources where incident light is normal to the surface across the wavelength range tested. There were multiple points along the spectrum where the penetration depth was less deep than the surrounding wavelengths. These reductions in penetration depth seem to correspond with peaks in the absorption spectra for the skin chromophores. The maximum penetration depth was at 810 nm and here 90% of the light reached approximately 1.2 mm. The MC model used by Finlayson *et al*., showed that changing the layer depths affected light penetration depth [80]. Similarly, Ash *et al*., used MC to determine the penetration depth in a skin model comprising of an 80 µm epidermis containing 5% melanin and approximately 3 mm dermis [81]. They also showed that penetration depth increased with wavelength, increasing from 500 µm to approximately 5.5 mm across wavelengths ranging from 300 nm – 750 nm. However, these models rely on accurate optical properties from the literature.

Using the diffusion equation, Phan *et al*., determined the penetration of 851 nm light into varying skin-types and showed that light travelled the furthest through subjects with the palest skin pigmentation (ranging from approximately 1.8 mm – 4.75 mm amongst the 10 subjects) [65]. Light penetration was least for subjects with the darkest skin pigmentation, ranging from approximately 1.85 mm to 2.5 mm amongst 5 subjects.

### 7.1 Determining transport mean free path to compare photon depth for published in-vivo and ex-vivo data

Here we calculate the transport mean free path (TMFP) as described in section 2 to determine the average distance over which a photon will travel before substantially changing its direction due to collisions with scattering particles in media consisting of the average scattering parameters collated from published results. TMFP is an important guide for imaging applications where it is necessary to focus light at a specific depth.

**Fig. 9.**
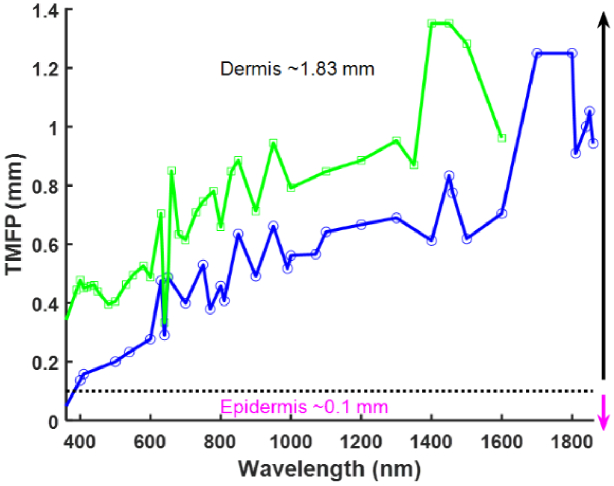
Comparison of average TMFP for published *ex-vivo* (blue) and *in-vivo* (green) data.

Using this calculation, the TMFP through *in-vivo* and *ex-vivo* skin using the published data was compared (Fig. 9) and shows that at a wavelength of 1400 nm the TMFP for *in-vivo* data is maximal (1.35 mm). The implications of using the values measured *in-vivo* is that light will be predicted to travel further into the skin compared to the values measured *ex-vivo* in the 400 nm – 1600 nm wavelength range. Because the TMFP is related to the reduced scattering coefficient, peaks in the scattering graph (Fig. 6) correspond with troughs in the TMFP graphs and reasons that scattering may be lower in the *in-vivo* situation were discussed in section 6. From Fig. 9 it can been seen that at wavelengths greater than 400 nm all the data reviewed here suggest that light will keep traveling in a well-defined direction well into the dermis layer. However, if one uses the *in-vivo* data recorded at 1400 nm, light would be expected to travel 1.38 mm in to the skin before it became diffuse compared to 0.6 mm for the same wavelength using the *ex-vivo* data.

### 7.2 Comparison of the proportion of photons reaching different depths in the skin using in-vivo and ex-vivo published data

The transmission of light through the skin was approximated using Eq. (2) (section 2). Using the published data, the proportion of light transmitted through an epidermis of 0.1 mm and a dermis ranging from 0.1–1.83 mm was calculated to determine the percentage of photons reaching given depths within, or even beyond, the skin. The thickness of the epidermis and dermis used were taken from Meglinski *et al*., and used as an example of average skin layer depths [58].

**Fig. 10.**
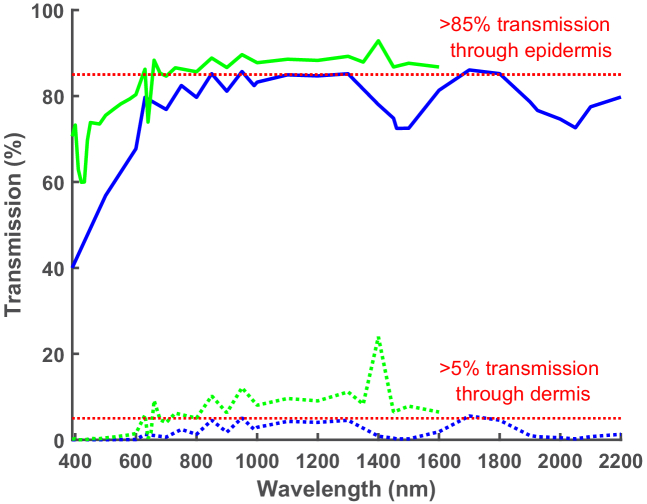
Comparison of transmission of light through *in-vivo* (green) and *ex-vivo* (blue) skin. Percentage epidermal transmission represented by solid lines, percentage of dermal transmission represented by dashed line. Epidermis = 0.1 mm thick; dermis = 1.83 mm thick.

Figure 10 shows that in general, transmission through in-vivo skin is predicted to be greater than ex-vivo across the spectrum measured. On average the difference in light transmission beyond the in-vivo and ex-vivo epidermis across the spectrum is approximately 11%, however this difference increases to 77% when comparing ex-vivo and in-vivo transmission through the epidermis and beyond the dermis. The lowest amount of light transmitted is predicted to be in the visible spectrum and this is probably due to melanin absorption of visible light in the epidermis. However, minimal transmission in this region is ∼60% using the in-vivo epidermis data compared with ∼42% when using the ex-vivo epidermis values. At these rates of transmission virtually no light is transmitted beyond the dermis. In general, the published data suggests that to achieve more than ∼5% transmission beyond the dermis more than ∼85% of light into the epidermis must be transmitted beyond it and ∼78% of light must be transmitted beyond the epidermis for more than ∼1% of light to be transmitted beyond the dermis.

Transmission through *in-vivo* skin peaks at 1400 nm, with ∼93% of the light into the epidermis being transmitted beyond it and ∼24% of this light being transmitted beyond the dermis. A corresponding peak in transmission is not observed for *ex-vivo* skin, in fact between 1300 nm and 1500 nm transmission decreased from ∼85% to ∼7 2% transmission trough the epidermis and ∼4.5% to ∼0.2% transmission beyond the dermis. This decrease in transmission may be attributed to water absorption of light in *ex-vivo* skin, due to rehydration during sample preparation and unrealistic water content. Maximal transmission through *ex-vivo* samples is at 1700nm. At this point TMFP is at its greatest and scattering and absorption troughs are coincident suggesting that this wavelength range might have the potential to provide deeper imaging and better resolution than at shorter wavelengths. Since the optical coefficients were only measured up to 1600 nm for the published *in-vivo* data conclusions on transmission at even longer wavelengths cannot be drawn however, it would be useful to determine the potential of longer wavelengths for increased transmission.

## 8. Summary and conclusions

To image non-invasively and deeply into the human body and potentially diagnose disorders, the skin is the first barrier to light. The aims of this review were to evaluate the variability amongst the *ex-vivo* and *in-vivo* optical properties of skin published in the literature, looking for trends in the reported values with wavelength, and to understand the controls required to produce a reliable set of skin optical properties. Our findings with regards to both aims can be found in the summary boxes at the start of this review. As shown for *ex-vivo* data, we report vast discrepancies in the optical properties of skin for measurements taken *in-vivo*, particularly with regards to scattering [8]. We looked to put these numbers in context by considering what they meant in terms of the amount of light able to reach the different layers within the skin and the maximum depth to which the light can be considering to be travelling predominantly in the forward direction (TMFP). The depth and proportion of light transmitted through the skin are important when developing optical technologies designed to image into the body or technologies to determine clinically useful information from the deeper skin layers and beyond. However, the huge variation in values reported in the literature makes producing reliable values for light transmission and TMFP at different wavelengths challenging.

The NIR wavelength range is commonly thought to provide the best potential to image deeply due to a reduction in scattering events and the presence of absorption troughs. Published *ex-vivo* and *in-vivo* absorption and scattering data are different, however absorption increases beyond 1300 nm for both. For both *in-vivo* and *ex-vivo* data melanin absorption is dominant up to 600 nm and water absorption is dominant at 1460 nm showing the importance of avoiding chromophore absorption peaks for diagnostic and imaging applications. However, absorption coefficients are much greater *ex-vivo* than *in-vivo* and this is probably due to sample preparation techniques and rehydration leading to unrealistic water content and greater absorption by water for *ex-vivo* samples. Beyond 1450 nm *in-vivo* reduced scattering increases, however, for *ex-vivo* measurements it steadily decreases between 1500 nm and 1800nm. This inconsistency in the published values has the potential to affect the design of deep imaging systems using longer wavelengths and needs to be investigated further. *Ex-vivo*, between 1600 nm – 1800nm, troughs in reduced scattering and absorption are coincidental with peaks in TMFP and percentage of light transmission suggesting that this wavelength range might have the potential for deeper imaging than at shorter wavelengths. Further investigation is required because it is difficult to draw useful conclusions regarding transmission beyond 1000 nm since there is little published data in this region, particularly *in-vivo*.

The published literature tells us that the optical properties determined are dependent upon multiple elements which cannot be ignored. It is clear that the published data available for skin in the *in-vivo* situation are variable and incomplete and a comprehensive dataset covering a broad set of wavelength measurements is required for optimising skin treatments and imaging. Variability is intrinsic, and a major contributor to the intrinsic variability in the *in-vivo* measurements is skin pigmentation. Some authors have stated skin pigmentation in their publications and it would be useful to evaluate the effect of this on optical properties with the aim of identifying wavelengths at which skin pigmentation becomes ‘invisible’ particularly when considering recent publications and potential inequality of healthcare [82–87]. However, that is not within the scope of this review. Other factors that would also need to be controlled for include measurement site, subject age and gender, measurement instrumentation and use of simulations that account for skin inhomogeneities.

In addition, changes in the polarisation state of light can be used to provide information about tissues, including the superficial layers of the skin, by characterising the depolarisation of light as it propagates [88,89]. This has been shown to be of importance for detecting skin cancers where the degree of depolarisation has allowed differentiation between cancerous and benign lesions [90,91]. In reality, what happens first, loss of ballistic photons due to scattering or depolarisation of light as it propagates, is dependent upon the size (essentially the *g* factor), shape and concentration of the scatterer [92]. For example, melanin has been shown to depolarise light [93]. Polarisation effects were generally ignored amongst the papers reviewed here and so we are unable to draw conclusions. However, we suggest that the polarisation of the light source used for characterisation should be included when reporting the optical properties of the skin in the future.

Despite the deeply scattering nature of the human tissues, light has been used for multiple biological applications including cancer diagnosis and studying cellular changes like apoptosis and it can provide structural information deriving from tissue boundaries, cells and organelles with OCT currently imaging to depths of approximately 1 mm in the skin [4]. If scattered light can be detected at depths greater than those currently achieved using OCT, and the scattering corrected for, using approaches such as wavefront shaping, new forms of optical imaging could provide important medical imaging tools in the clinic [94]. These new techniques would complement existing methods and provide useful insight into diseases such as cancer and arthritis.

In our opinion, the main role of *ex-vivo* data is looking at skin layers in more detail; the optical properties for each of these layers are likely to compare poorly to the *in-vivo* case, but relationships between the optical properties for each skin layer may be of use. We suggest that published *ex-vivo* optical coefficients are unlikely to be useful for aiding the development of deep imaging techniques and that reliable *in-vivo* data are required.

## Data Availability

Data underlying the results presented in this paper are available in Refs. [12,15,21,37,46,49,50,52–61,63–65].
